# Epidemiological and Clinical Characteristics of Respiratory Syncytial Virus Infection in Children in Hangzhou (2022–2023)

**DOI:** 10.3390/pathogens14060603

**Published:** 2025-06-19

**Authors:** Qin-Rui Lai, Xiao-Li Chu, Ying-Ying Chen, Wei Li, Ya-Jun Guo, Shi-Qiang Shang

**Affiliations:** Department of Clinical Laboratory, The Children’s Hospital, Zhejiang University School of Medicine, Hangzhou 310052, Chinachweige@zju.edu.cn (W.L.)

**Keywords:** respiratory syncytial virus, epidemiology, children, infection

## Abstract

Human respiratory syncytial virus (HRSV) is a highly contagious RNA virus that causes respiratory infections, especially in children. This study evaluated the impact of COVID-19 control measure changes on HRSV infection patterns in Hangzhou by comparing epidemiological and clinical characteristics. We conducted a retrospective analysis of 12,993 pediatric nasopharyngeal swab samples from children with acute respiratory infections at The Children’s Hospital of Zhejiang University School of Medicine. These samples, collected between February 2022 and January 2024, were screened for HRSV and 12 other respiratory pathogens using capillary electrophoresis technology. From February 2022 to January 2023, the HRSV positivity rate was 7.06%. In 2023, it increased to 9.26%. The highest positivity rates were in infants aged 0–6 months and children aged 6 months to 1 year. Coinfections were most common with rhinovirus in 2022 and *Mycoplasma pneumoniae* in 2023. HRSV positivity rates were low from April to September 2022, peaking in December. In 2023, the peak occurred from April to September. Symptoms ranged from mild to severe pneumonia, with higher hospitalization rates in children with underlying conditions. The study revealed significant changes in HRSV infection rates following pandemic restriction relaxations, emphasizing the need for the early identification and prevention of severe cases.

## 1. Introduction

Human respiratory syncytial virus (HRSV), newly classified as *Orthopneumovirus hominis*, is an enveloped, negative-sense single-stranded RNA virus belonging to the *Pneumoviridae* family [1]. It is a widely circulating pathogen among humans. HRSV was initially isolated from chimpanzees in 1956 [2] and subsequently obtained from infants with severe lower respiratory tract diseases [3]. The HRSV genome comprises 10 genes encoding 11 proteins. The small hydrophobic protein (SH), attachment glycoprotein (G), and fusion protein (F) play pivotal roles in the lifecycle of HRSV [4].

HRSV infection is a seasonal disease with varying onset, resolution, duration, and peak times annually. Seasonality also differs by geographic region. In the Northern Hemisphere, the season typically spans from November to April. In China, HRSV epidemics typically occur in winter and spring [5,6]. HRSV infection can manifest as various clinical symptoms, ranging from benign upper respiratory tract diseases to severe lower respiratory tract infections (LRTIs), as well as potential long-term effects such as childhood asthma. HRSV is most closely associated with acute LRTI [7]. Globally, HRSV ranks as the second leading cause of death from pediatric LRTIs [8,9]. This results in narrowed airways, increased resistance, and distal air trapping or atelectasis [10]. It is estimated that HRSV causes acute respiratory infections in approximately 4.03% of Chinese children aged 0–60 months annually. Among pathogens of non-severe and severe community-acquired pneumonia in children, HRSV accounts for 17.8% and 21.30%, respectively, ranking first in both categories [8,11].

In China, the control of COVID-19 spread through extensive public health interventions led to significant changes in human behavior, subsequently affecting the transmission of other seasonal respiratory viruses, including HRSV [12]. In 2020 and 2021, there was a sharp decline in global HRSV and influenza infections [13]. Nationwide lockdowns and other measures used to slow the spread of COVID-19 prevented the transmission of seasonal pathogens, reducing opportunities for people to build immunity to these microbes and leading to an increased susceptibility to respiratory viruses in the population, with more reports of HRSV outbreaks in hospitals [14,15]. In the post-pandemic era, it appears to be one of the most significant respiratory viral pathogens in children, causing considerable morbidity and hospitalization costs. At the end of 2022, the rate of hospitalization related to HRSV infection for infants and children was higher than that in the same period from 2010 to 2020 [16,17]. In resource-limited settings, HRSV-related deaths predominantly occur in full-term infants, whereas in resource-rich countries, most deaths are observed in children with underlying risk factors [17].

To evaluate how dynamic non-pharmaceutical interventions (NPIs) during the COVID-19 pandemic (2022–2024) reshaped HRSV epidemiology in Hangzhou, we compared infection patterns across the following two phases: a strict control phase (February 2022–January 2023) and a transitional phase (February 2023–January 2024) marked by the gradual relaxation of restrictions. This period captures the immediate effects of shifting containment strategies, including the nationwide policy shift in December 2023. Our analysis focuses on identifying HRSV’s resurgence dynamics and clinical severity trends under evolving public health measures.

## 2. Methods and Materials

### 2.1. Sample Collection

A retrospective analysis was conducted on outpatient and inpatient cases of acute respiratory infections caused by HRSV infection at The Children’s Hospital of Zhejiang University School of Medicine (Hangzhou, Zhejiang, China) from February 2022 to January 2024. Nasopharyngeal swabs were collected within 48 h of symptom onset from pediatric patients (age < 18 years) presenting with symptoms of respiratory infections, including sore throat, stuffy nose, runny nose, cough and phlegm, fever, or shortness of breath. To avoid duplicate counts, only the first HRSV-positive sample from each patient was included; subsequent samples within 30 days were excluded unless separated by a negative test result. Oropharyngeal samples were preserved in 2.5 mL of specialized viral transport medium (KaiBiLi, Hangzhou, China).

### 2.2. Laboratory Assay

#### 2.2.1. Nucleic Acid Extraction and Amplification

Automatic nucleic acid extraction was performed using nucleic acid extraction reagents (Health, Ningbo, China). During the procedure, a specific volume of samples (including positive and negative controls, provided by Juno Genomics, Hangzhou, China) was aspirated and combined with 2 μL of RT-PCR internal control for extraction. A multiplex detection kit for 13 respiratory pathogens (fluorescence PCR-capillary electrophoresis method) was utilized for pathogen detection (Health, Ningbo, China), including influenza A virus (FluA) subtypes H1N1 and H3N2, influenza B virus (FluB), human parainfluenza virus (HPIV), adenovirus (AdV), *Mycoplasma pneumoniae* (Mp), *Chlamydophila pneumoniae* (Cp), human bocavirus (HBoV), coronavirus (CoV), human rhinovirus (HRV), human metapneumovirus (HMPV), and HRSV. Detection was performed via capillary electrophoresis, a technology solution offered by Juno Genomics (Hangzhou, China). The respiratory 13-plex premix and RT-PCR enzyme solution were mixed. Fifteen microliters of the mixture were aliquoted into individual 8-tube racks and centrifuged for 10 s. The RT-PCR amplification program strictly followed the manufacturer’s instructions.

#### 2.2.2. Capillary Electrophoresis

Mix 90 µL of AB Master Mix with 10 µL of the standard sample and combine 9 µL of the AB Master Mix with 1 µL of PCR product to prepare the electrophoresis samples. Add 10 µL of these mixtures into each well of a 96-well plate. Load the sample plate into the CE2400 automatic capillary electrophoresis instrument for electrophoretic separation. Assess the infection status by comparing the peak heights of the specific loci of the PCR products in each well with the peak heights of the standard sample.

The combination of multiplex fluorescent PCR and capillary electrophoresis offers significant advantages in genetic testing and nucleic acid analysis. This technique enables the simultaneous detection of multiple targets, making it especially suitable for identifying groups of pathogens in cases of co-infection. It not only conserves resources and reduces the financial burden on patients but also enhances detection efficiency and cuts costs. In clinical sample analysis, capillary electrophoresis fragment analysis demonstrates higher effectiveness in minimizing false positive and false negative results compared to qPCR methods [18].

### 2.3. Data Collection

Patient information for both inpatients and outpatients who tested positive for HRSV, including patient name, sex, age, and primary clinical symptoms, was collected. The clinical data were classified into mild and severe pneumonia categories, and other analyses were conducted according to the “Diagnosis and Treatment Guidelines for Community-Acquired Pneumonia in Children (2019 Edition) [19]”.

### 2.4. Statistical Analysis

Data analysis included medians, chi-square tests, and Fisher’s exact tests for group comparisons. All tests were two-sided, with *p*-values < 0.05 considered significant. Analysis was conducted using IBM SPSS Statistics 27.

## 3. Results

### 3.1. Epidemiology Characteristics

**Figure 1:**
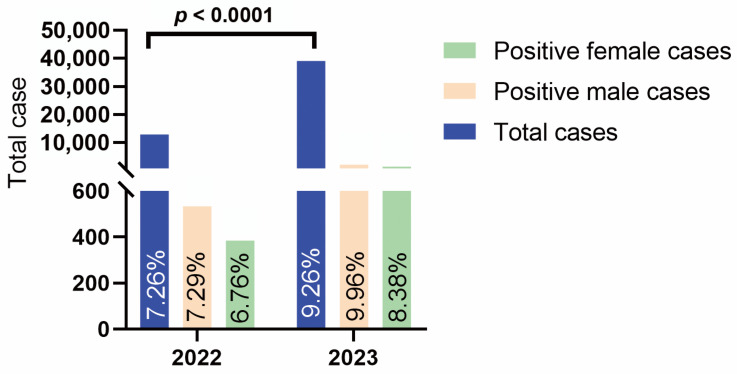
Total HRSV tests and positive cases by gender in 2022 and 2023.

As shown in Figure 1, from February 2022 to January 2023, we collected a total of 12,993 samples, of which 917 (7.06%, 917/12,993) tested positive for HRSV. Within this group, 533 patients were male (7.29%, 533/7311), and 384 were female (6.76%, 384/5682). From February 2023 to January 2024, the number of samples increased to 39,019, with 3612 (9.26%, 3612/39,019) testing positive for HRSV, including 2152 males (9.96%, 2152/21,597) and 1460 females (8.38%, 1460/17,422). The comparison of the overall incidence rates between the two periods revealed a significant difference (*p* < 0.0001), indicating a significant difference in the overall incidence rates between 2022 and 2023.

**Figure 2:**
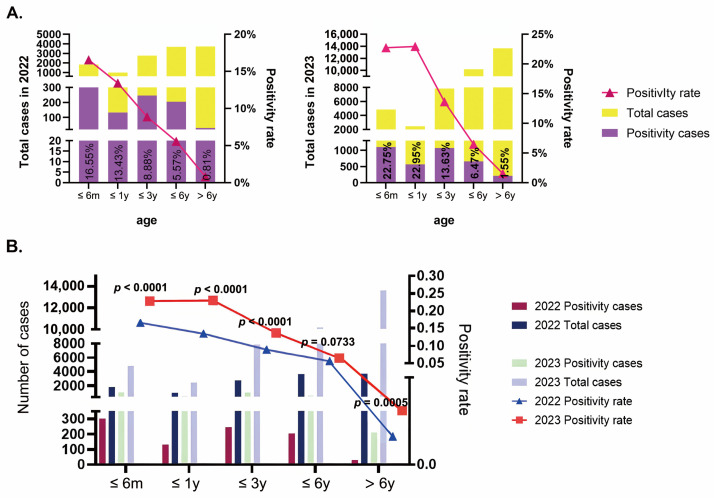
Age-specific HRSV positivity rates and annual trends: (**A**) positive rates of HRSV by age group; (**B**) comparison of 2022 and 2023 positivity cases and rates across different age groups.

A comparative analysis of HRSV-positive rates across different age groups in 2022 and 2023 was conducted (Figure 2A). Patients were stratified into the following mutually exclusive age categories: ≤6 months, ≤1 year (>6–12 months), ≤3 years (>12–36 months), ≤6 years (>36–72 months), and >6 years (>72 months). The results indicated a notable increase in positive rates across all age groups. Specifically, the positive rate for infants aged 0–6 months increased from 16.55% (303/1831) in 2022 to 22.75% (1102/4844) in 2023. Similarly, the positive rate for children aged ≤1 year increased from 13.43% (133/990) to 22.95% (568/2475). Although children aged ≤3 years had relatively lower positive rates, there was still an increasing trend, with rates of 8.88% (246/2771) in 2022 and 13.63% (1071/7857) in 2023. The positive rates for children aged ≤6 years were 5.57% (205/3681) in 2022 and 6.47% (659/10,184) in 2023. Children >6 years old exhibited the lowest positive rates, with 0.81% (30/3720) in 2022 and 1.55% (212/13,659) in 2023. The highest positive rate in 2022 was observed in the ≤6 month age group, whereas in 2023, it was highest in the ≤1 year age group. Statistical analysis revealed significant differences in positive rates among the different age groups. Further comparison between 2022 and 2023 (Figure 2B) revealed that, except for the 3–6 years group, which showed no statistically significant difference (*p* = 0.0733), the differences between the same age groups in different years were statistically significant.

**Figure 3:**
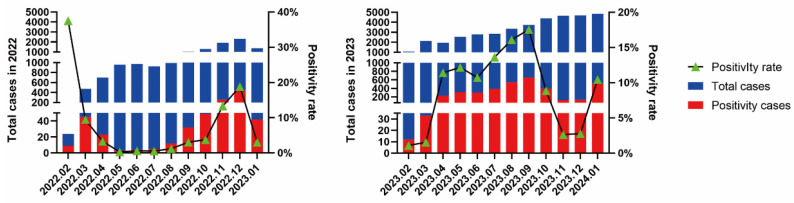
Monthly positive detection rates of HRSV.

The monthly positive detection rates of HRSV in 2022 and 2023 exhibited distinct epidemic trends (Figure 3). In 2022, HRSV-positive rates remained low from April to September, with an increasing trend starting in October and reaching a peak in December. In contrast, in 2023, the HRSV epidemic peaked from April to September, with detection rates declining from October to December. Concerning coinfection cases, the most common coinfection pathogen in HRSV-positive cases in 2022 was HRV, with a coinfection rate of 3.49% (32/917). In 2023, Mp became the primary coinfecting pathogen, with a coinfection rate of 6.42% (232/3612). The specific types of coinfections and their positive rates are presented in Table 1.

### 3.2. Clinical Characteristics

**Figure 4:**
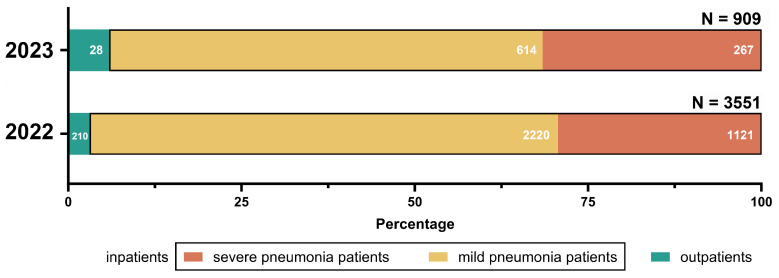
Distribution of HRSV-positive cases by clinical setting.

Through the hospital information system, we collected all HRSV-induced pneumonia case information (Figure 4). Notably, minor discrepancies between total HRSV-positive samples and analyzed clinical cases resulted from the exclusion of patients with incomplete medical records or guardians declining anonymized data sharing under ethical protocols. According to diagnostic and treatment guidelines, from February 2022 to January 2023, there were 28 (3.08%, 28/909) outpatient cases and 881 (96.92%, 881/909) inpatient cases of HRSV-positive patients, including 267 (30.31%, 267/881) cases of severe pneumonia and 614 (69.69%, 614/881) cases of mild pneumonia. From February 2023 to January 2024, the number of HRSV-positive outpatient cases increased to 210 (5.91%, 210/3551), with 3341 (94.09%, 3341/3551) inpatient cases, including 1121 cases (33.55%, 1121/3341) of severe pneumonia and 2220 cases (66.45%, 2220/3341) of mild pneumonia.

**Figure 5:**
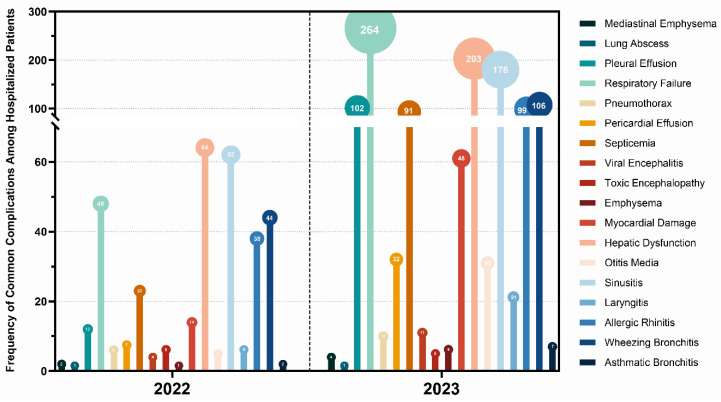
Common complications of HRSV infection. **Note**: single patient may present multiple complications, comorbidities, or coinfections; counts represent the total number of instances, not individual patients.

Symptoms of HRSV-induced pneumonia include fever, runny nose, and cough. While the majority of these infections are mild, severe cases may progress to significant pneumonia or respiratory failure, potentially leading to complications of varying degrees (Figure 5).

**Figure 6:**
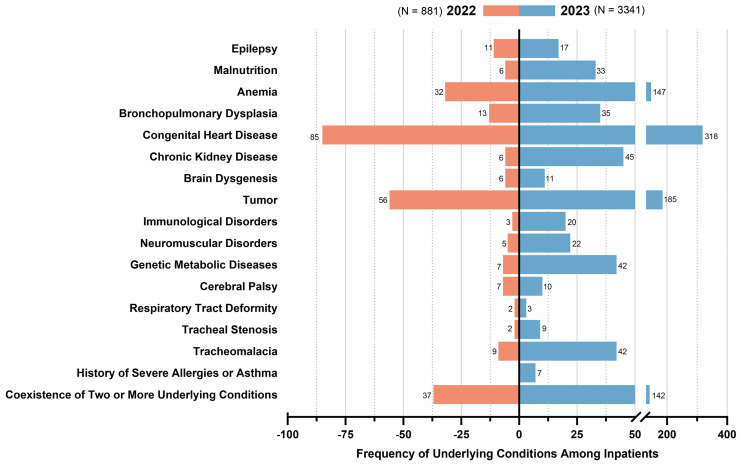
Frequency of underlying medical conditions. **Note**: single patient may present multiple complications, comorbidities, or coinfections; counts represent the total number of instances, not individual patients.

In 2022, 213 (26.22%, 213/881) of the hospitalized patients had underlying medical conditions. In 2023, 781 (22.38%, 781/3341) of the hospitalized patients had underlying medical conditions. Common underlying conditions are listed in Figure 6.

**Figure 7:**
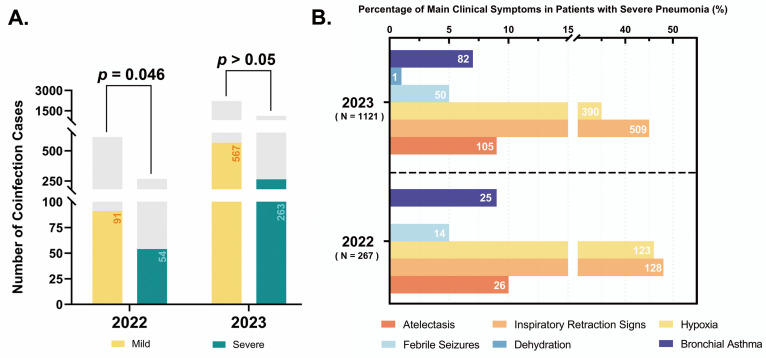
Clinical characteristics of HRSV-positive inpatients from 2022 to 2023: (**A**) coinfection rates in mild versus severe pneumonia; (**B**) dominant clinical symptoms in severe pneumonia. **Note**: single patient may present multiple complications, comorbidities, or coinfections; counts represent the total number of instances, not individual patients.

We classified the clinical data into mild and severe pneumonia for further analysis. In 2022, among the 614 mild HRSV infection cases, 91 had coinfections with other pathogens, while, among the 267 severe cases, 54 were diagnosed with coinfections. In 2023, of the 2220 mild cases, 567 had confirmed coinfections, and among the 1122 severe cases, 263 were found to have coinfections (Figure 7A). To better characterize the clinical progression of severe pneumonia, we systematically documented the predominant clinical manifestations observed in severe pneumonia patients (Figure 7B).

## 4. Discussion

HRSV is a highly infectious agent that causes lower respiratory tract infections [12], with the potential to trigger a wide range of illnesses, ranging from mild to severe, in children. This has garnered considerable international concern. We found that the HRSV positivity rate in our hospital increased from 7.06% (917/12,993) in 2022 to 9.26% (3612/39,019) in 2023, indicating a statistically significant increase. It is important to note that we collected all eligible samples each month based on strict clinical standards. In only February 2022, the HRSV positivity rate might be affected by fewer samples. For other periods, positivity rate variations were not due to more samples collected. Our analysis confirmed that increased sample volume did not correlate with changes in positivity rates over time. The analysis of the age distribution revealed that positivity rates for HRSV increased across all age groups in 2023 compared to those in 2022, except for the 3–6 years old age group, where the increase was not statistically significant (*p* = 0.0733). All other age groups exhibited statistically significant increases. The HRSV epidemic is driven by a complex interaction between climate, host, and virus. The overall positivity rate and the increased positivity rates across different age groups in this study are likely due to the various levels of NPIs and the dynamic zero-COVID policy implemented in China for nearly three years, which reduced the transmission of many viral respiratory pathogens. The impact of NPIs on the infection rates of respiratory pathogens has been confirmed in multiple studies [13,20]. With the relaxation of pandemic restrictions by the end of 2023, the reduced natural immunity exposure to other respiratory viruses led to an increase in susceptible populations, resulting in a surge of HRSV cases following the easing of control measures. Modeling studies in South Africa, The Netherlands, and Japan have also predicted potential HRSV epidemics following the lifting of restrictions [14]. Similar surges in HRSV cases were observed in other regions of China, such as Ningbo [15] and Shijiazhuang [7], after the relaxation of policies. Our findings indicate that the highest positivity rates among children are concentrated in the 0–1 year age group, which is consistent with previous studies [8,10]. The highest positivity rate in infants aged 0 to 1 year may be associated with their immature immune systems [16,17]. Notably, maternal vaccination can confer passive immune protection through the transplacental transfer of high antibody titers, providing defense against infection during the first months of life [21]. Recent clinical trials have shown that maternal RSV vaccination reduces medically attended severe RSV-associated lower respiratory tract infections by 81.8% within 90 days postpartum [22]. The persistently high positivity rate in the 0–1 year children observed in our study suggests that, despite the presence of maternally derived antibodies, naturally acquired immunity appears insufficient to fully prevent infections. This finding showed the critical need to enhance protection in this vulnerable population through active immunization strategies, such as maternal vaccination. In terms of seasonality, previous studies have indicated that the transmission and prevalence of HRSV exhibit distinct seasonal patterns, typically beginning to rise in the autumn and peaking in the winter [23]. However, data from 2023 indicate that the peak of the epidemic occurred earlier than in the previous year, with the peak period occurring from April to September and a decline in detection rates observed from October to December. Sun et al. also observed this seasonal trend change in HRSV in 2023, which they speculated might be related to the interruption of influenza outbreaks [15]. Additionally, some studies have reported that the seasonal patterns of HRSV are not well defined and are unstable, with outbreaks occurring almost year-round [24]. This variability may be attributable to the influence of meteorological factors, such as temperature and relative humidity, on the seasonal patterns of HRSV [25,26]. In terms of coinfections, RV was the most common coinfecting pathogen in 2022, and it is also the most frequently observed virus to coinfect with HRSV [27]. However, in 2023, MP emerged as the primary coinfecting pathogen. This may be related to its high prevalence in the community [28] and shared risk factors for transmission with HRSV.

Through our clinical symptom analysis of pneumonia cases caused by HRSV, we found that, in addition to common respiratory complications, extrapulmonary complications such as liver function injury are also prevalent. Studies have shown that HRSV can directly invade the liver, even leading to HRSV-associated hepatitis [29], indicating the necessity of liver function testing in infected children. One notable feature of HRSV-induced pneumonia is the signs and symptoms associated with increased airway resistance [30]. In our research, severe pneumonia caused by HRSV predominantly presented clinically with symptoms such as atelectasis, inspiratory retraction, and hypoxemia. Additionally, patients frequently exhibited typical radiographic characteristics, such as hyperinflation and patchy infiltrates on chest X-rays, which further support the clinical diagnosis and assist in assessing the severity of the condition [31]. These clinical presentations are crucial for identifying and preventing the progression of severe pneumonia. Furthermore, the presence of underlying medical conditions significantly increases the severity of HRSV infections, which is consistent with findings from previous studies [32,33]. In our study, the impact of coinfections on clinical severity showed different statistical significances between 2022 and 2023. Notably, the observed increase in severe pneumonia cases in 2023 (33.55% vs. 30.31% in 2022) may be primarily attributed to immunity debt resulting from prolonged NPIs during the COVID-19 pandemic. Previous research has also debated the correlation between HRSV and clinical severity. Several studies have reported no difference in clinical outcomes among coinfected patients or observed increased ICU admission risks only when patients were coinfected with specific viruses such as hMPV [34,35]. In contrast, other studies have indicated that disease severity increases in patients with isolated HRSV infections [36]. Therefore, some researchers hypothesize that, in cases of coinfection, the severity is primarily determined by the HRSV infection itself rather than the coinfecting virus itself [34]. These findings suggest a highly complex role for HRSV in coinfections.

This study has several limitations. First, our cases were primarily from Hangzhou, with a few from other areas within the province, which may not represent the overall provincial situation. Further monitoring studies across multiple locations are needed to better elucidate HRSV’s epidemiological characteristics in Zhejiang Province. Second, HRSV isolates are classified into two main antigenic types (A and B), both of which are associated with varying disease severity [37]. Therefore, further genetic subtyping of HRSV is warranted. Despite these limitations, our study analyzed the epidemiological characteristics of HRSV during 2022–2023, revealing significant changes in infection rates following the relaxation of pandemic restrictions. By statistically analyzing clinical case data, this research further aims to facilitate the early identification of severe cases, thereby assisting in the prevention and progression of severe illnesses.

## Figures and Tables

**Table 1:** Positivity rates for different coinfection types.

Year	Coinfection Virus Types	Number of Cases [N (%)]
**2022** **(N = 153)**	HRV	32 (20.92)
	HPIV	28 (18.3)
	Mp	28 (18.3)
	HBoV	22 (14.37)
	AdV	10 (6.54)
	CoV	6 (3.92)
	HMPV	5 (3.27)
	AdV + Mp	4 (2.61)
	Cp	3 (1.96)
	HRV + Mp	3 (1.96)
	HRV + HBoV	2 (1.31)
	FluA H1N1	1 (0.65)
	FluA H3N2	1 (0.65)
	HRV + HPIV	1 (0.65)
	HRV + FluB	1 (0.65)
	HBoV + HPIV	1 (0.65)
	HPIV + CoV	1 (0.65)
	HBoV + FluA H3N2	1 (0.65)
	Mp + HPIV	1 (0.65)
	HRV + Mp + Cp	1 (0.65)
	HRV + Mp + HPIV + HBoV + HMPV	1 (0.65)
**2023** **(N = 923)**	Mp	232 (25.14)
	HRV	211 (22.86)
	HPIV	124 (13.43)
	HMPV	49 (5.31)
	AdV	31 (3.36)
	HBoV	30 (3.25)
	HRV + Mp	29 (3.14)
	CoV	27 (2.93)
	HPIV + HRV	26 (2.82)
	HPIV + Mp	22 (2.38)
	FluA H3N2	21 (2.28)
	FluB	18 (1.95)
	HBoV + Mp	9 (0.98)
	AdV + Mp	9 (0.98)
	HRV + HMPV	7 (0.76)
	HMPV	6 (0.65)
	AdV + HRV	6 (0.65)
	HBoV + HRV	5 (0.54)
	Cp	4 (0.43)
	HPIV + HMPV	4 (0.43)
	CoV + Mp	4 (0.43)
	FluA H3N2 + Mp	4 (0.43)
	HRV + CoV	3 (0.33)
	HPIV + CoV	3 (0.33)
	HPIV + AdV	3 (0.33)
	FluA H1N1	2 (0.22)
	FluB + Mp	2 (0.22)
	HPIV + HBoV	2 (0.22)
	HPIV + Cp	2 (0.22)
	HRV + HMPV	2 (0.22)
	HBoV + HRV + Mp	2 (0.22)
	HRV + Cp	1 (0.11)
	H1N1 + HPIV	1 (0.11)
	HMPV + Cp	1 (0.11)
	AdV + HBoV	1 (0.11)
	AdV + CoV	1 (0.11)
	AdV + HMPV	1 (0.11)
	Flu B + HMPV	1 (0.11)
	HPIV + HRV + Mp	1 (0.11)
	HPIV + HRV + HMPV	1 (0.11)
	HPIV + HBoV + HRV	1 (0.11)
	HPIV + HBoV + HMPV	1 (0.11)
	HPIV + AdV + HRV	1 (0.11)
	HPIV + AdV + Mp	1 (0.11)
	FluA H3N2 + HRV	1 (0.11)
	FluA H3N2 + HMPV	1 (0.11)
	FluA H3N2 + Flu B	1 (0.11)
	AdV + HRV + Mp	1 (0.11)
	AdV + HMPV + Mp	1 (0.11)
	Flu B + HMPV + Mp	1 (0.11)
	HPIV + HBoV + HRV + Mp	1 (0.11)
	HPIV + HBoV + HRV + HMPV	1 (0.11)
	FluA H3N2 + HBoV + HRV	1 (0.11)
	FluA H3N2 + HBoV + Mp	1 (0.11)
	FluA H3N2 + AdV + HRV	1 (0.11)

## Data Availability

The data supporting this study’s findings are available from the corresponding author upon reasonable request.
